# Self-extracellular RNA acts in synergy with exogenous danger signals to promote inflammation

**DOI:** 10.1371/journal.pone.0190002

**Published:** 2017-12-20

**Authors:** Frederik Noll, Jonas Behnke, Silke Leiting, Kerstin Troidl, Gustavo Teixeira Alves, Holger Müller-Redetzky, Klaus T. Preissner, Silvia Fischer

**Affiliations:** 1 Institute of Biochemistry, Medical School, Justus-Liebig-University, Giessen, Germany; 2 Max-Planck-Instiute for Heart and Lung research, Bad Nauheim, Germany; 3 Department of Vascular and Endovascular Surgery, University Hospital Frankfurt, Frankfurt, Germany; 4 Department of Infectious Diseases and Pulmonary Medicine, Charité – Universitätsmedizin Berlin, Berlin, Germany; University of the Pacific, UNITED STATES

## Abstract

Self-extracellular RNA (eRNA), released from stressed or injured cells upon various pathological situations such as ischemia-reperfusion-injury, has been shown to act as an alarmin by inducing procoagulatory and proinflammatory responses. In particular, M1-polarization of macrophages by eRNA resulted in the expression and release of a variety of cytokines, including tumor necrosis factor (TNF)-α or interleukin-6 (IL-6). The present study now investigates in which way self-eRNA may influence the response of macrophages towards various Toll-like receptor (TLR)-agonists. Isolated agonists of TLR2 (Pam2CSK4), TLR3 (PolyIC), TLR4 (LPS), or TLR7 (R848) induced the release of TNF-α in a concentration-dependent manner in murine macrophages, differentiated from bone marrow-derived stem cells by mouse colony stimulating factor. Here, the presence of eRNA shifted the dose-response curve for Pam2CSK4 (Pam) considerably to the left, indicating that eRNA synergistically enhanced the cytokine liberation from macrophages even at very low Pam-levels. The synergistic activation of TLR2 by eRNA/Pam was duplicated by other TLR2-agonists such as FSL-1 or Pam3CSK4. In contrast, for TLR4-agonists such as LPS a synergistic effect of eRNA was much weaker, and was not existent for TLR3-, or TLR7-agonists. The synergistic eRNA/Pam action was dependent on the NFκB-signaling pathway as well as on p38MAP- and MEK1/ERK-kinases and was prevented by predigestion of eRNA with RNase1 or by antibodies against TLR2. Thus, the presence of self-eRNA as alarming molecule sensitizes innate immune responses towards pathogen-associated molecular patterns (PAMPs) in a synergistic way and may thereby contribute to the differentiated outcome of inflammatory responses.

## Introduction

The innate immune system protects against infectious microbes by the recognition of PAMPs, which serve to detect pathogens on the host cell surface or in endosomes by pattern recognition receptors (PRRs) like Toll-like receptors (TLRs), Nucleotide-binding oligomerization domain-containing protein-1 (NOD1)-like receptors, mannose receptor, or retinoic acid-inducible gene-1-like receptors that initiate proper host defense mechanisms [1,2]. In addition to PAMPs, a series of endogenous danger-associated molecular patterns (DAMPs) are recognized by PRRs, thereby serving as body´s own alarm signals under conditions of stress or injury [3–5]. Among such DAMPs are heat shock proteins, high mobility group box 1 protein (HMGB-1), aged matrix proteins, S100 proteins, as well as nucleic acids. These usually intracellular factors acquire extracellular functions when released from cells by either passive or active processes [6]. Thus, DAMPs function as „alarmins”to alert the body about danger or disease by triggering inflammation, dendritic cell maturation, and stimulate the immune response resulting in the release of cytokines, which in turn can augment the local inflammatory environment [7]. Many DAMPs (like PAMPs) are recognized by PRRs in a promiscuous fashion by TLR2, TLR4, or the receptor for advanced glycation end products (RAGE) [3,8]. Binding of DAMPS or PAMPs to TLRs activates either myeloid differentiation factor 88 (MyD88)—dependent or Toll/interleukin-1 receptor domain containing adaptor inducing interferon β (TRIF)—dependent signaling pathways, resulting in the activation of transcription factors like JNK or NF-κB, followed by the release of cytokines including TNF-α, interleukins (IL)-1β, or IL-6 [9,10].

Self eRNA released under pathological conditions such as myocardial ischemia-reperfusion injury has been described to activate the TLR3-Trif pathway and to induce inflammatory responses in cardiomyocytes and immune cells by TLR7-signaling, which were both abolished by pretreatment of RNA samples with RNase, but not DNase [11,12]. Furthermore, RAGE was identified to bind eRNA and to enhance its cellular uptake into endosomes leading to an enhanced TLR response [13]. Accordingly, in previous studies we have characterized self-extracellular RNA (eRNA) as a new alarmin, which induces prothrombotic, permeability-increasing, and inflammation-promoting responses in immune and vascular cells [14–19]. In these investigations, eRNA was used as single agonist at rather high concentrations and for extended incubation periods with different *in vitro* cell culture systems [14,17]. In the present study, the inflammatory potential of eRNA in the presence of additional PAMPs and DAMPS was tested, whereby eRNA was found to serve as potent adjuvant, particularly for TLR2 ligands to enhance their proinflammatory potential in a synergistic manner. Thus, the presence of self-DAMPs such as eRNA during situations of microbial infection may very well potentiate the outcome of inflammation, and counteracting regimen such as administration of RNase1 could be of potential therapeutic value.

## Materials and methods

### Cell culture

Primary bone marrow derived macrophages (BMDM) were obtained from bone marrow cells (BMCs), which were flushed from the bones of C57Bl/6 mice. Animal care and all experimental procedures were performed in strict accordance to the German and National Institutes of Health Animal Legislation Guidelines and were approved by the local animal care and use committees (Regierungspräsidium Giessen). BMCs were cultured in DMEM medium (Gibco) containing 10% fetal calf serum (FCS, Gibco) and differentiated by mouse-colony stimulating factor, which is secreted by L929 cells and is used in the form of L929-conditioned medium. Under these conditions, BMDM proliferate and differentiate into a homogenous population of mature BMDMs after 1 week. The efficiency of the differentiation was assessed by fluorescence-activated cell sorting (FACS) analysis of antigens F4/80, CD68 and CD11b, reaching a cellular purity of about 90%. To stimulate BMDM in future experiments, 2 x 10^6^ cells/ml RPMI were used.

The following agents at the indicated concentrations were used for cell stimulations: lipopolysaccharide (LPS) from Sigma-Aldrich (München, Germany), Pam2CSK4, Pam3CSK4, FSL-1, polyIC, R848, and MAb-mTLR2 from InvivoGen (Toulouse, France), PD98059 and SB203580 from Calbiochem (Merk, Darmstadt, Germany), Bay 11–7082 from Enzo Life Sciences (Lörrach, Germany), and TNF-α receptor antagonist WP9QY from Santa Cruz (Heidelberg, Germany). Prior to stimulation, cells were washed once with phosphate-buffered saline (PBS) and incubated for the indicated time periods in cell culture medium containing the different agents at the indicated concentrations. Stimulation of cells with Pam2CSK4/eRNA mixtures was performed after preincubating both agents in PBS for 1 h at 37°C.

### Isolation and quantification of RNA and DNA

Total cellular RNA and DNA were isolated from macrophages or from confluent cultures of fibroblasts using an extraction kit (Peqlab, Erlangen, Germany) or the DNAzol reagent (Invitrogen, Groningen, The Netherlands). eRNA, which was used to stimulate cells, was purified from confluent cultures of fibroblasts by using the TRIzol^™^ reagent, followed by precipitation with isopropanol and resuspending the RNA in RNase-free water. Subsequently, the dissolved RNA was additionally purified using the TRIzol LS reagent in accordance with instructions of the manufacturer (Thermo Fisher Scientific, Darmstadt, Germany). To isolate eRNA from cell supernatants, fibroblasts or epithelial cells were stimulated with small, non-toxic concentrations of the pore-forming bacterial toxin pneumolysin (200 ng/ml; friendly supplied by Professor Tim Mitchell, Birmingham, USA) for 1 h. Cell-supernatants were first centrifuged for 5 min at 200 x g to remove cells and cell debris. To prevent degradation of RNA, RNase inhibitor (4 U/ml, RNasin, Invitrogen) was added and samples were concentrated using centricon tubes (cut off 10 kD; Millipore) that were centrifuged at 3400 x g for 12 min at 4°C and subsequently washed with autoclaved sterile water. Same amounts of lysis buffer (peqGOLD total RNA kit from Peqlab) were added to the concentrated cell supernatants and RNA was isolated in accordance with the instructions of the manufacturer. Total RNA and DNA were quantified using a NanoDrop 2000 (Thermo Fisher Scientific). Quality of RNA and DNA was confirmed by electrophoresis on 1% agarose gels followed by ethidium bromide staining or by using the Agilent 2100 bioanalyzer and the Agilent RNA 6000 Nano Kit (Agilent Technologies, Konstanz, Germany). The quality of RNA isolated from cell supernatants or lysates was identical, but the amount of RNA isolated from cell supernatants was rather small (4–7 μg from 6 x 10^6^ cells) compared to cell lysates. Purity of RNA was confirmed by performing the endotoxin test using the PierceTM LAL chromogenic endotoxin quantification kit (Thermo Fisher Scientific). The concentration of endotoxin found in RNA preparations used for the stimulation of cells was always < 0.1 endotoxin Units (EU)/ml, which correlates to 0.01 ng/ml of endotoxin. These concentrations of endotoxin did not induce any inflammatory response.

### Binding of biotinylated RNA to proteins

Wells of a microtiter plates were coated each with 50 μl solution of platelet factor 4 (PF4), TLR2, CD14, CD36 (each from PeproTech, London, UK), or bovine serum albumin (BSA) (10 μg/ml each) in 100 mM sodium carbonate (pH 9.5) at 4°C for 20 h. Wells were washed and blocked for 2h with Tris-buffered saline (TBS) containing 3% BSA. Different concentrations of biotinylated RNA (0. 78–50 μg/ml), prepared from RNA using the Psoralen-PEO-Biotin reagent (Pierce, Rockford, IL), were allowed to bind at 22°C for 2 h followed by 3 times washing with TBS. Bound biotinylated RNA was detected using peroxidase-conjugated streptavidin (Dako, Glostrup, Denmark) and the immunopure TMB (3,3’,5,5’-tetramethylbenzidine) substrate kit (Pierce) by measuring the reaction products at 450 nm. Binding of biotinylated RNA to PF4 and BSA was considered as positive and negative controls, respectively [20].

### Quantitative real-time PCR (qPCR)

Following treatment of BMDM with various agonists as indicated in the legends of the corresponding figures, cells were washed twice with PBS, lysed, and RNA was isolated with the total RNA extraction kit (Peqlab). For qPCR analysis, 1 μg of RNA was reverse-transcribed using the High-Capacity cDNA Reverse Transcription Kit (Applied Biosystems, Carlsbad, CA, USA) and DNA amplification was performed with a StepOne Plus cycler (Applied Biosystems) in a reaction volume of 10 μl using the SensiMix Sybr Kit (Bioline, Luckenwalde, Germany) with 50 pmol of each primer. To avoid amplification of genomic DNA, primers were designed to span exon-exon junctions. The qPCR was performed under the following conditions: an initial denaturation step at 95°C for 8.5 min followed by 45 cycles, consisting of denaturation (95°C, 30 s), annealing (60°C, 30 s) and elongation (72°C, 30 s). Melt curve analysis was performed to control specific amplification. Results were normalized to the expression levels (E) of actin and expressed as the ratio of E(target)/E(Actin). The following mouse primers were used: TNF-α forward ACTGAACTTCGGGGTGATCG, TNF-α reverse TGGTTTGTGAGTGTGAGGGTC, IL-6 forward CTCTGCAAGAGACTTCCATCCA, IL-6 reverse TTGTGAAGTAGGGAAGGCCG, actin forward CGCGAGCACAGCTTCTTTG, actin reverse CGTCATCCATGGCGAACTGG, IL-1β forward GGATGAGGACATGAGCACCT, IL-1β reverse GGAGCCTGTAGTGCAGTTGT, MCP-1 forward AAGCTGTAGTTTTTGTCACCAAGC, MCP-1 reverse GACCTTAGGGCAGATGCAGTT.

### TNF-α- and IL-6-ELISA

Macrophages were treated for different time periods with various agents as indicated in the corresponding figure legends. TNF-α- and IL-6- ELISA was performed using the commercially available kit from eBioscience (Frankfurt, Germany).

### Statistical analysis

Each experiment was repeated at least three times. The mean values are presented as mean ± standard error of the mean (SEM). The data of each figure were analysed using one way analysis of variance (ANOVA) followed by multiple comparisons between different groups by the Bonferroni Multiple Comparison Test. All statistical analyses were performed using GraphPad Prism (version 5.0). A value p < 0.05 was considered to represent statistical significance.

## Results

### Synergistic function of eRNA and PAMPs on the activation of TLR-2

**Fig 1:**
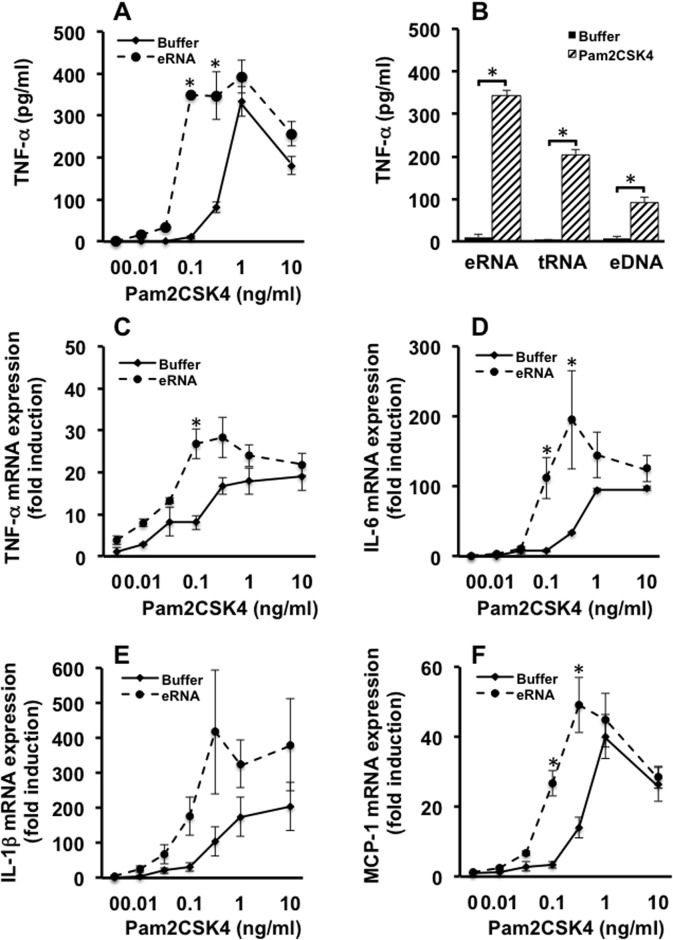
Sensitizing function of eRNA on TLR2 activation by Pam2CSK4. Macrophages were treated for 2 h with different concentrations of Pam2CSK4 in the presence of eRNA (10 μg/ml) or buffer (A, C-F) or a fixed concentration of Pam2CSK4 (0.1 ng/ml) or buffer in the presence of eRNA, tRNA, or eDNA (each 10 μg/ml) (B). Prior to cell stimulation, nucleic acids and Pam2CSK4 were preincubated for 1 h at 37°C. Cell supernatants were analyzed for the release of TNF-α by ELISA. The mRNA expression of TNF-α (C), IL-6 (D), IL1β (E), and MCP-1 (F) was assessed from macrophage lysates by qRT-PCR. Values are expressed as mean ± SEM; N = 3–12; *P < 0.05 between groups.

To investigate the influence of eRNA on the PAMP-mediated release of TNF-α from macrophages, cells were treated with different concentrations of the TLR-2/6 agonist Pam2CSK4 (Pam) in the absence or presence of eRNA. Pam alone at concentrations ≥ 0.3 ng/ml increased the TNF-α release in a concentration-dependent manner after two hours of stimulation. However, in the presence of eRNA, a significant shift of the dose-response curve to the left was observed, resulting in a dramatic increase of TNF-α release already at more than tenfold lower Pam concentrations (0.01–0.03 ng/ml). Only at Pam-concentrations ≥ 1 ng/ml, the difference between the TNF-α release induced by Pam alone or in combination with eRNA was no more significant (Fig 1A). Accordingly, the mRNA expression of inflammatory cytokines, such as TNF-α, IL-6, IL-1β and monocyte chemoattractant protein-1 (MCP-1) were significantly elevated by Pam concentrations ≥ 0.03 ng/ml in the presence of eRNA (Fig 1C–1F). Not only eRNA, consisting mostly of ribosomal RNA, but also tRNA promoted the release of TNF-α up to 70-fold in the presence of suboptimal concentrations of Pam (0.1 ng/ml), which had no stimulatory function on its own. eDNA exhibited moderate synergistic activity together with Pam (Fig 1B). The synergistic activation of TLR2 by eRNA/Pam was only expressed if both agonists were preincubated for at least 1h prior to cell stimulation. Predigestion of eRNA with RNase1 reduced the release of TNF-α at the Pam concentration of 0.1 ng/ml to 39 ± 11% compared to the cytokine release induced by eRNA/Pam mixture, confirming the involvement of eRNA in the synergistic activation of TLR2. However, activity of eRNA was independent of its binding to TLR2 or to cofactors of TLRs such as CD36 and CD14 as was demonstrated by a binding assay of biotinylated RNA to each of these proteins (S1 Fig).

**Fig 2:**
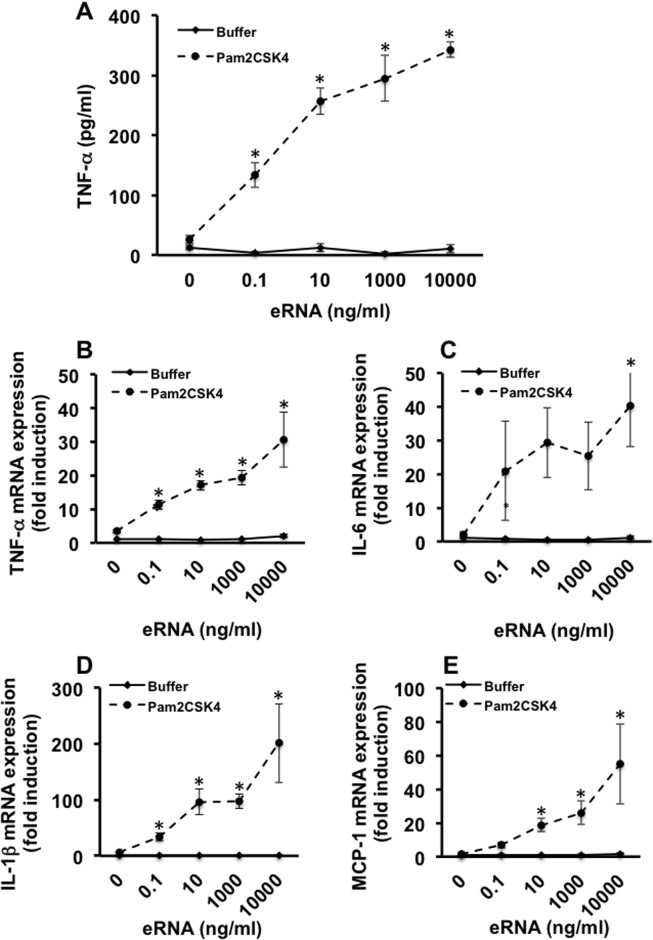
Sensitizing function of different concentrations of eRNA on TLR2 activation. Macrophages were treated for 2 h with different concentrations of eRNA in the presence of Pam2CSK4 (0.1 ng/ml) or buffer. Prior to cell stimulation, eRNA and Pam2CSK4 were preincubated for 1 h at 37°C. Supernatants of cells were analyzed for the release of TNF-α by ELISA (A). mRNA expression of TNF-α (B), IL-6 (C), IL1β (D), and MCP-1 (E), was assessed from cell lysates by qRT-PCR. Values are expressed as mean ± SEM; N = 3; *P < 0.05 between groups.

The dose-response curve of eRNA in the presence of low, by itself inactive Pam concentration (0.1 ng/ml), revealed that the presence of increasing eRNA concentrations resulted in a significant concomitant increase in the release of TNF-α. In particular, the ratio of TNF-α release determined in the presence versus the absence of Pam increased from 30-fold at 0.1 ng/ml eRNA to maximal 200-fold at 10 μg/ml eRNA (Fig 2A). Accordingly, the mRNA expression of TNF-α, IL-6, IL-1β and MCP-1 reflected the same profile of the dose-response curve of eRNA in the presence of 0.1 ng/ml Pam (Fig 2B–2E).

**Fig 3:**
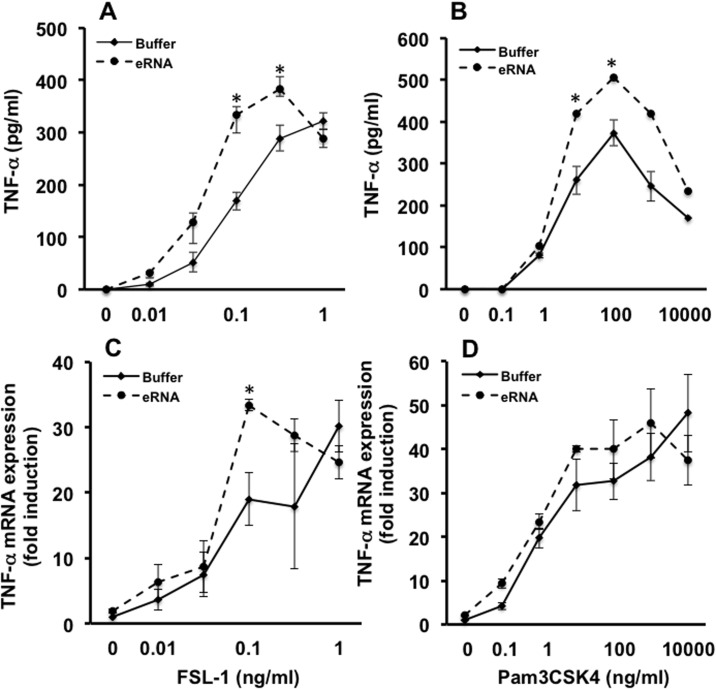
Sensitizing function of eRNA on TLR2 activation by TLR2-agonists FSL-1 and Pam3CSK4. Macrophages were treated for 2 h with different concentrations of FSL-1 (A,C) or Pam3CSK4 (B,D) in the presence of eRNA (10 μg/ml) or buffer. Prior to cell stimulation, eRNA was preincubated with FSL-1 or Pam3CSK4 for 1 h at 37°C. Supernatants of cells were analyzed for the release of TNF-α by ELISA (A,B) and mRNA expression of TNF-α was determined from cell lysates by qRT-PCR (C,D). Values are expressed as mean ± SEM; N = 3–8; *P < 0.05 between groups.

Similar results were obtained when the TLR2/6 agonist FSL-1 instead of Pam was used: At concentrations 0.03 ng/ml– 0.3 ng/ml of FSL-1, eRNA significantly increased the release of TNF-α protein from macrophages compared to the cytokine release induced by FSL-1 alone, whereas the differences in the induction of TNF-α mRNA expression became only obvious at a FSL-1 concentration of 0.1 ng/ml (Fig 3A and 3C). By using the TLR2/1 agonist Pam3CSK4, the sensitizing effects of eRNA on TLR2 activation became significant only at higher concentrations of Pam3CSK4 (> 1 ng/ml) compared to the previously mentioned TLR2/6 agonists, whereas TNF-α mRNA expression was not changed in the presence of eRNA (Fig 3B and 3D). The mRNA expression of IL-6, IL-1β and MCP-1 exhibited the same profile of the dose-response curve of FSL-1 and Pam3CSK4 in the presence of eRNA (10 μg/ml) as was observed for mRNA expression and release of TNF-α (S2 Fig).

### Time-dependent TLR2 activation by combined agonists

**Fig 4:**
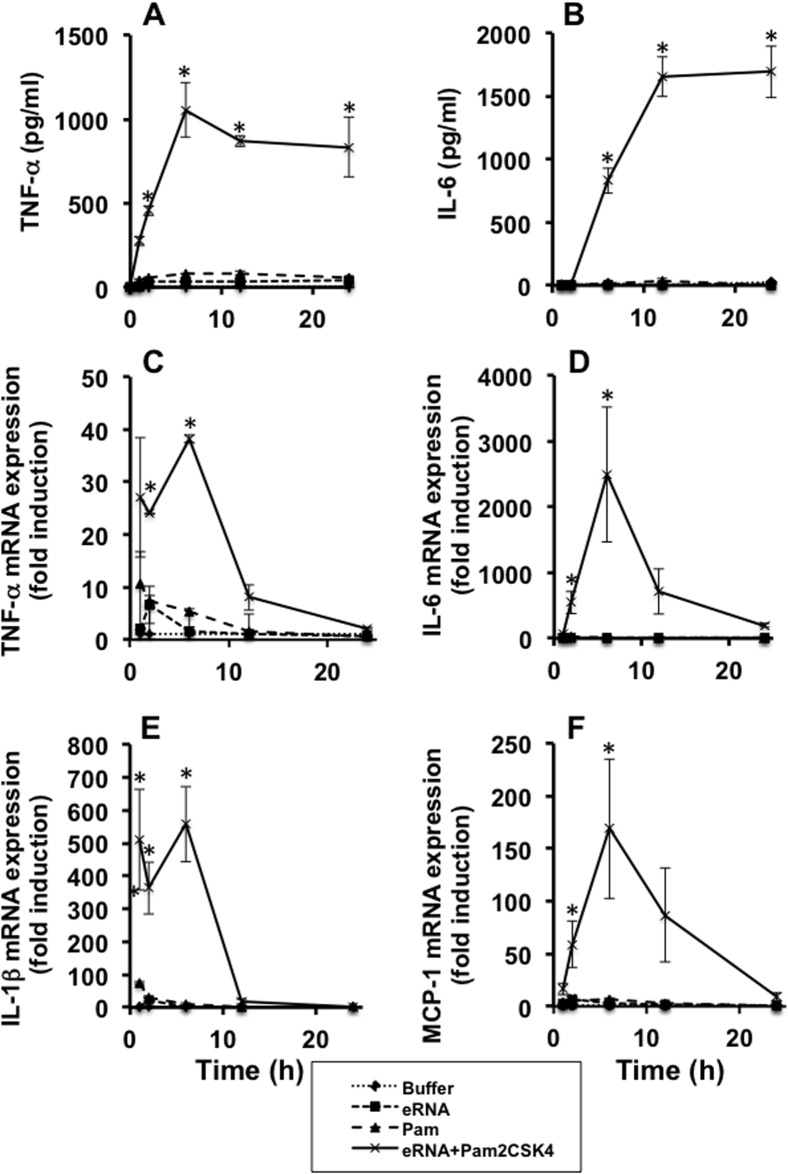
Time-dependent cytokine expression from macrophages treated with eRNA/Pam2CSK4. Following preincubation of eRNA (10 μg/ml) and Pam2CSK4 (0.1 ng/ml) for 1 h at 37°C, macrophages were treated with these combined agonists (eRNA+Pam2CSK4), eRNA, Pam, or buffer for different time periods as indicated. Supernatants of cells were analyzed for the release of TNF-α (A) and IL-6 (B) by ELISA. mRNA expression of TNF-α (C), IL-6 (D), IL-1β (E), and MCP-1 (F) was assessed from cell lysates by qRT-PCR. Values are expressed as mean ± SEM; N = 3; *P < 0.05 versus buffer-treated group.

The sensitizing effect of eRNA on the Pam-mediated TNF-α release could be further increased by prolonged exposure of macrophages towards these agonists. For example, upon exposure of cells towards eRNA/Pam for longer time periods, the mRNA expression of TNF-α IL-6, IL-1β and MCP-1 increased up to 6 h, but thereafter, it decreased or totally declined, respectively (Fig 4C–4F). Using the same experimental setting, the release of TNF-α protein increased up to 6 h and then remained unchanged, whereas the release of IL-6 protein only started after 6 h of treatment (Fig 4A and 4B).

To exclude that autocrine effects of TNF-α were involved in the activation of macrophages, cell stimulation was performed in the presence of a TNF-α-receptor antagonist. The TNF-α release induced by eRNA/Pam in the presence of the TNF-α-receptor blocker was unchanged after 2 h of treatment, whereas after 6 h, the cytokine release was significantly reduced by the TNF-α-receptor antagonist (S3 Fig). Thus, activation of macrophages was performed only for 2 h to prevent any interfering effects by TNF-α itself.

### Signaling pathways involved in TLR2 activation by combined eRNA/Pam treatment

**Fig 5:**
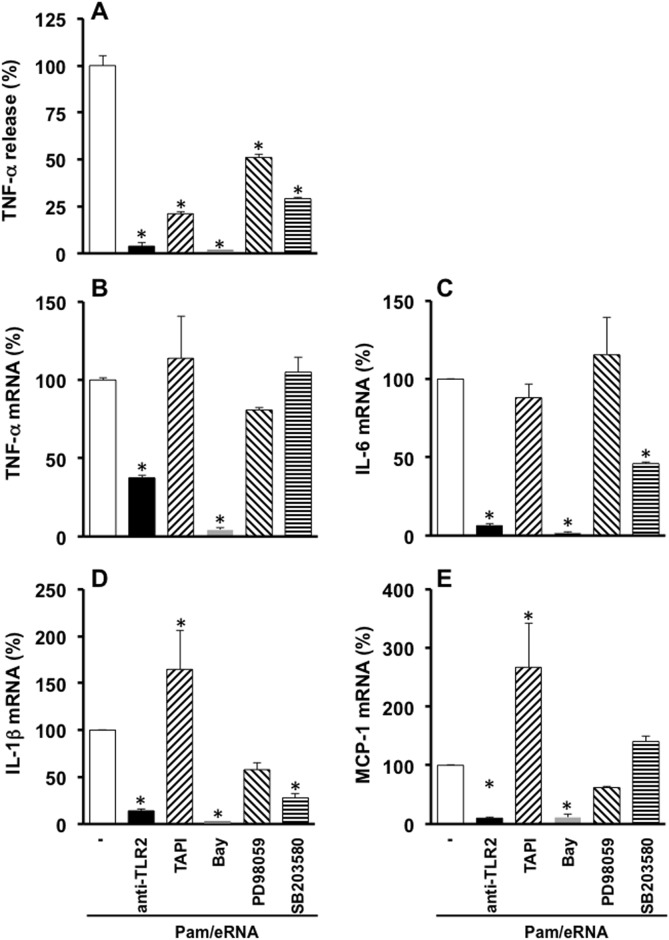
Intracellular signaling involved in the synergistic eRNA/Pam2CSK4-mediated TLR2 activation. Macrophages were pretreated with buffer, neutralizing antibodies against TLR2 (1 μg/ml), TAPI (10 μg/ml), Bay (100 μM), PD98059 (20 μM), or SB203580 (10 μM) for 30 min, and stimulated for 2 h with eRNA (10 μg/ml)/Pam2CSK4 (Pam, 0.1 ng/ml), which were preincubated for 1 h at 37°C. Supernatants were analyzed for the release of TNF-α by ELISA (A). Cell lysates were used for the quantification of mRNA expression of TNF-α (B), IL-6 (C), IL-1β (D), and MCP-1 (E) by qRT-PCR. TNF-α release and mRNA expression induced by eRNA/Pam in the absence of additives were set to 100%. Values are expressed as mean ± SEM; N = 3; *P < 0.05 versus values from eRNA/Pam-treated cells in the absence of additives.

To confirm the involvement of TLR2 in the macrophage response towards the combined agonists eRNA/Pam, a TLR2 neutralizing antibody was shown to abrogate not only the eRNA/Pam-induced increase in TNF-α protein but also the mRNA expression of TNF-α, IL-6, IL-1β, and MCP-1 (Fig 5A–5E). Moreover, the release of active TNF-α protein, which depends on the activation of the sheddase ADAM17, was abolished by the ADAM17-inhibitor TAPI (Fig 5A). Since the activation of TLR2 involves engagement of the NFκB-pathway in any case, mRNA expression and protein release were totally blocked by the NFκB pathway inhibitor Bay. In contrast, blockade of MAPkinases 42/44 and 38 by PD98059 and SB203580, respectively, partially reduced the release of TNF-α protein but did not affect mRNA expression of this cytokine (Fig 5A and 5B) and had variable effects on mRNA expression of IL-6, IL-1β, and MCP-1 (Fig 5C–5E). No change in the cytokine mRNA expression or protein release was observed if these inhibitors were used in the absence of TLR2 agonists. While the expression of TLR2 itself was slightly increased by eRNA or Pam alone and eRNA/Pam as well, other TLRs such as TLR3, 4 or 7 were not significantly affected in their mRNA expression upon exposure to either one of these agonists (S1 Supplement).

### Influence of eRNA on the activation of TLR 3, 4, and 7

**Fig 6:**
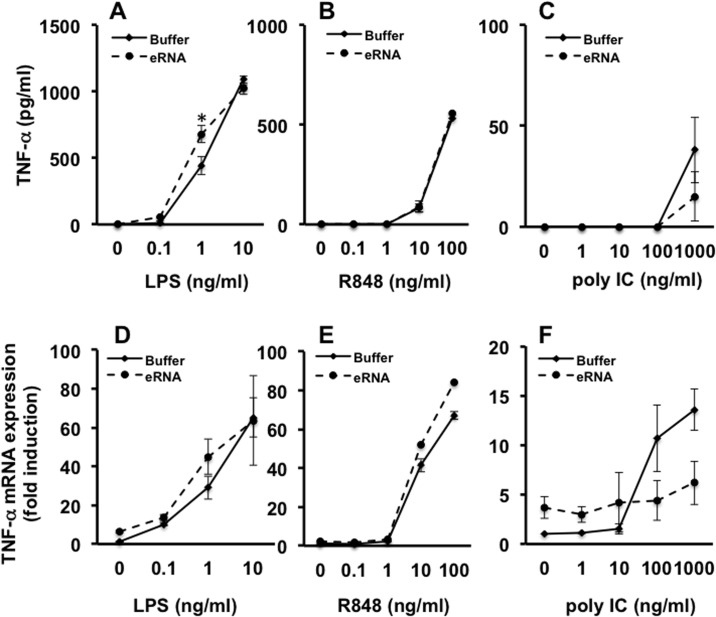
Influence of eRNA on the activation of TLRs. Macrophages were treated for 2 h with different concentrations of LPS (A), R848 (B), or poly IC (C) in the presence of eRNA (10 μg/ml) or buffer. Prior to cell stimulation, eRNA was preincubated with each agonist for 1 h at 37°C and supernatants of cells were analyzed for the release of TNF-α by ELISA (A). mRNA expression of TNF-α (B), IL-6 (C), IL-1β (D), and MCP-1(E) was assessed from cell lysates by qRT-PCR. Values are expressed as mean ± SEM. N = 3; *P < 0.05 between groups.

eRNA only slightly induced the activation of TLR4 by LPS, since at 1 ng/ml LPS (but not below or above this concentration) the release of TNF-α was moderately but significantly increased by eRNA (Fig 6A) without any remarkable effect on mRNA expression (Fig 6D). In contrast, eRNA had no influence on the activation of TLR7 and TLR3 by R848 and polyIC, as measured by the release of TNF-α (Fig 6B, 6C, 6E and 6F).

## Discussion

In the present study, self-eRNA was identified as a synergistic cofactor for Pam2CSK4, a typical TLR2 agonist, to strongly enhance macrophage activation. The combined action of eRNA and Pam was not apparent for endosomal TLRs such as TLR3, TLR 7, or TLR 8, which all recognize non-self RNA. However, under conditions of high abundance of self RNAs resulting from cell damage, defects in the RNA-degradation machinery, or after bindung and cellular uptake of RNA into endosomes, such self RNAs may activate endosomal PRRs [13,21]. The combined action of eRNA/Pam resulted in a substantially elevated mRNA expression and release of cytokines in murine bone marrow-derived macrophages, whereas each single agonist at the same concentration was ineffective. Thus, the synergistic action of eRNA and Pam demonstrates a novel example of pathogen recognition and sensitization by host cells that is markedly influenced by DAMPs/alarmins and may only be observed in situations where a certain degree of cell stress or trauma with the release of eRNA has occured.

Another well-studied example of synergistic pathogen sensitization is CD14, the co-receptor for TLR4 in the recognition of LPS and the internalization of TLR2 ligands such as lipoteichoic acid or Pam3CSK4 [22–24]. Moreover, CD14 also binds to triacylated lipopeptides facilitating their recognition by TLR2/TLR1 complexes [22]. Another case is CD36, which mainly acts as a sensor of diacylated lipopeptides that become recognised by TLR2/TLR6 [25]. The large difference between these surface-associated host proteins and eRNA is that the latter becomes released as DAMP upon cell stress or damage anywhere in the organism and thereby may fulfill a universal role as alarmin and sensitizer of TLR2-dependent cell responses.

As eRNA does not directly bind to CD14 or CD36 and also not to TLR2 itself, it is fair to assume that polymeric eRNA may serve as a scaffolding factor and could directly interact with multiple basic Pam molecules to ensure their enhanced recognition by TLR2. This would explain that Pam promotes cell activation at very low concentrations only in the presence of eRNA. An equivalent type of mechanism was observed in an experimental system with eRNA and vascular endothelial growth factor (VEGF) as interaction partners, since only in the presence of eRNA very low doses of VEGF could operate to induce endothelial cell signaling [16]. In the present study it became obvious that the synergistic action of eRNA on TLR2 activation was dependent on the respective TLR2 ligand, whereby Pam2CSK4 showed a much higher synergistic response in comparison to FSL-1, although both ligands are known to bind to TLR2/TLR6 heterodimers. Accordingly, synergistic activities of eRNA might rely on structure- and charge-dependent interactions with TLR2 ligands to facilitate TLR-activation. Moreover, eRNA required preincubation with Pam2CSK4 prior to exposure towards macrophages to promote a synergistic TLR2-dependent response and eRNA digestion with RNase1 prior to the addition of Pam completely abolished eRNA/Pam-induced cytokine release.

Non-self bacterial DNA or RNA are protected from degradation, for example by their capsids, until they are released within the endo-lysosomal compartment of host cells, whereas self extracellular nucleic acids are degraded by nucleases, unless they are protected in nucleoprotein complexes or by association with cell membranes [26]. Nevertheless, self nucleic acids may only reach endo-lysosomes and PRRs if bound to proteins or manipulated by e.g. transfection agents. For example, the antimicrobial peptide LL37 or HMGB1 can bind and protect RNA from degradation and shuttle it into endo-lysosomal compartments [27,28], while self-RNA can be experimentally modified by transfection agents such as lipofectamin to reach intracellular compartments [11,12]. The latter, however, does not reflect the natural situation that was experimentally challenged in the present study. Since the degradation of eRNA by RNase1 was not changed in the presence of Pam, the synergistic action of eRNA and Pam on TLR2 activation appears not to be related to eRNA protection against RNase1. The cellular activity of eRNA might also depend on heterodimerization of TLRs and the type of TLR involved, as synergistic functions of eRNA were much stronger on agonists recognizing the TLR2/TLR6- compared to the TLR2/TLR1-heterodimer. Moreover, only a minor eRNA-dependent synergistic effect was seen for LPS in the activation of the TLR4 axis, and no influence was recognized for the typical RNA-receptors TLR3, TLR7 or TLR8. At high concentrations of TLR3 agonist, poly IC, eRNA even seems to decrease the poly IC-induced TNF-α expression slightly, but not significantly. Ligands of TLR3, like negatively charged double-stranded RNA, are quite different from ligands of TLR2 such as Pam containing more basic amino acids, which might change the binding modes of these ligands to their receptors in the presence of eRNA. However, these results indicate that activities of DAMPs, which were identified to amplify the inflammatory response of PAMPs, differ appreciably regarding the activation of particular PRRs [29,30].

During longer periods of treatment, the continuous TNF-α release from macrophages was apparently due to autocrine stimulation as well, since blockade of the macrophage TNF-α receptor signaling resulted in diminished TNF-α-release. Activation of the inflammasome leading to the caspase-1-dependent secretion of IL-1β seems not to be involved in the synergistic action of eRNA/Pam on TLR2 activation, as no release of IL-1β was detectable even after treatment of macrophages for longer time intervals up to 24 h [31].

Accordingly, a former study demonstrated DAMP-sensing of PRRs, whereby activation of endosomal TLRs by self-eRNA resulted in the uptake of eRNA into endosomes in a sequence-independent manner by binding to the receptor for advanced glycation end-products (RAGE), which had been identified as a surface-expressed self-eRNA receptor [32]. Moreover, the DAMP HMGB1 has been recognized as binding partner and general sentinel for immunogenic nucleic acids, as the absense of HMGB1 severely impaired the activation of TLR3, TLR7, and TLR9 by their cognate nucleic acid ligands [28]. HMGB1 further plays an important role in sepsis by facilitating the transfer of LPS to CD14 to initiate a TLR4-dependent proinflammatory response [29]. Likewise, the extracellular matrix component biglycan can act as an endogenous ligand of TLR4 and TLR2 and thereby sensitizes cells towards LPS [33]. In accordance with these general mechanisms, we now describe eRNA as a sentinel for the activation of TLR2 by its cognate PAMP agonists to amplify the release of cytokines.

As cytokine-inducing activity of eRNA/Pam was inhibited by neutralizing antibodies against TLR2, Myd88-induced pathways are likely to be involved. Further signaling pathways required for the eRNA/Pam2SCK4-induced cytokine release involved the transcriptional activation of the NF-κB system, because blockade of this pathway fully abrogated the mRNA-expression as well as the release of TNF-α protein in macrophages. In contrast, MAPkinase inhibitors only blocked the TNF-α release but not the mRNA expression, indicating the involvement of these MAPkinases in the activation of ADAM17, which is necessary for the shedding reaction of membrane-bound pro-TNF-α to release active soluble TNF-α [34]. As TNF-α is liberated by the activation of ADAM17, this cytokine was already detected after 2 h treatment with eRNA/Pam (both ineffective when used alone), whereas the release of IL-6 protein was detectable only after 6 h stimulation. Accordingly, the shedding of active TNF-α protein but not the mRNA expression of TNF-α, induced by eRNA/Pam, was blocked by TAPI, an inhibitor of ADAM17. Surprisingly, the presence of TAPI significantly increased the mRNA expression of IL-1β and MCP-1 indicating a self-regulatory mechanism to compensate the missing TNF-α release.

Several studies demonstrated TLR activation even by isolated endogenous DAMPs, whereas the corresponding signaling pathways remain largely unknown. The leucine-rich repeat motif of TLRs has been shown to be involved in the binding of PAMPs, but there is evidence that DAMPs require further cofactors or coreceptors for the optimal activation of TLRs and their association with diverse signaling routes [33–37]. A recent study even indicates that the reported cytokine expression in response to putative endogenous ligands may in fact be due to PAMP contaminations [38,39].

In summary, in situations of cellular stress or upon infection, the DAMP eRNA serves a prominent cofactor role in facilitating PAMP-mediated activation of TLR2. Only in a combined fashion, eRNA and TLR2-agonists promote activation of TLR2 on macrophages, resulting in appreciable cytokine mRNA expression and release, whereas each single agonist at the given concentration did not induce any inflammatory response. Thus, it is proposed that the degree of tissue damage with the appearance of eRNA provides the appropriate condition for PAMP-dependent TLR2 activation. As a consequence, chronic inflammatory diseases like rheumatoid arthritis, cancer, or atherosclerosis may depend on such perpetuated proinflammatory responses uncovered in this study. In fact, eRNA was shown to be responsible for the development of such diseases in preclinical experimental animal models, whereby RNase1 administration resulted in a significant blockade of the inflammatory outcome and in considerable tissue protection [40–42].

## Supporting information

S1 Fig Synergistic activities of eRNA/Pam2CSK4 on TLR2 activation: Time-dependent preincubation. (A) Macrophages were treated with buffer (Control), Pam2CSK4 (Pam, 0.1 ng/ml), eRNA (10 μg/ml), or eRNA/Pam preincubated for different time intervalls prior to the treatment of cells for 2h. (B) Macrophages were treated with buffer (Control), 1h preincubated eRNA/Pam2CSK4 (eRNA/Pam), or eRNA predigested for 1h with RNase1 followed by 1h preincubation with Pam2CSK4 (eRNA(hydr.)/Pam). Supernatants of cells were analyzed for the release of TNF-α. Values are expressed as mean ± SEM. N = 3; *P < 0.05 between groups. (C) Binding of biotinylated RNA to immobilized PF4, TLR2, CD36, and CD14 was performed in a solid binding assay and data are corrected for unspecific binding to BSA. Data represent the mean ± SEM; N = 3.
(TIFF)

S2 Fig Sensitizing function of eRNA on TLR2 activation by TLR2-agonists FSL-1 and Pam3CSK4. Macrophages were treated for 2 h with different concentrations of FSL-1 (A-C) or Pam3CSK4 (D-F) in the presence of eRNA (10 μg/ml) or buffer. Prior to cell stimulation, eRNA and FSL-1 or Pam2CSK4 were preincubated for 1 h at 37°C. mRNA expression of IL-6, IL-1β, and MCP1 was assessed from cell lysates by qRT-PCR. Values are expressed as mean ± SEM; N = 3–8; *P < 0.05 between groups.
(TIFF)

S3 Fig Time dependent TNF-α release from macrophages treated with eRNA/Pam2CSK4. Macrophages were treated with eRNA (10 μg/ml)/Pam2CSK4 (0.1 ng/ml) (preincubated for 1 h at 37°C) for 2 h and 6 h in the presence of TNF-α receptor antagonist or buffer. Supernatants were analyzed for the release of TNF-α. Values determined in the absence of TNF-α receptor antagonist were set to 100%. Values are expressed as mean ± SEM; N = 3; *P < 0.05 between groups.
(TIFF)

S4 Fig Influence of eRNA and Pam2CSK4 on the expression of Toll-like receptors. Macrophages were treated with eRNA (10 μg/ml), Pam2CSK4 (0.1 ng/ml), buffer, or the preincubated mixture for 2 h. mRNA expression of TLR2, TLR3, TLR4 and TLR7 was assessed from cell lysates by qRT-PCR. The expression of buffer-treated cells was set to one, and the data represent fold induction ± SEM; N = 3.
(TIFF)

S1 Supplement (DOCX)

## Abbreviations

ADAM: a disintegrin and metalloprotease
BMC: bone marrow cell
BMDM: bone marrow derived macrophages
DAMP: danger-associated molecular patterns
eDNA: extracellular DNA
ELISA: enzyme-linked Immunosorbent Assay
eRNA: extracellular RNA
FACS: fluorescence-activated cell sorting
FCS: fetal calf serum
HMGB1: high mobility group box 1 protein
hydr.: hydrolyzed
IL: interleukin
LPS: lipopolysaccharid
MCP-1: monocyte chemoattractant protein-1
NOD1: nucleotide-binding oligomerization domain-containing protein-1
Pam: Pam2CSK4
PAMP: pathogen-associated molecular patterns
PBS: phosphate-buffered saline
PRR: pattern recognition receptor
qPCR: quantitative real-time PCR
RAGE: receptor for advanced glycation end products
SEM: standard error of the mean
TLR: Toll-like receptor
TNF-α: tumor necrosis factor-α
VEGF: vascular endothelial growth factor
