# Supplementary figures and images for: Self-extracellular RNA acts in synergy with exogenous danger signals to promote inflammation
Source: PLoS One. 2017 Dec 20;12(12):e0190002. doi: 10.1371/journal.pone.0190002 (PMC5738100; doi:10.1371/journal.pone.0190002)

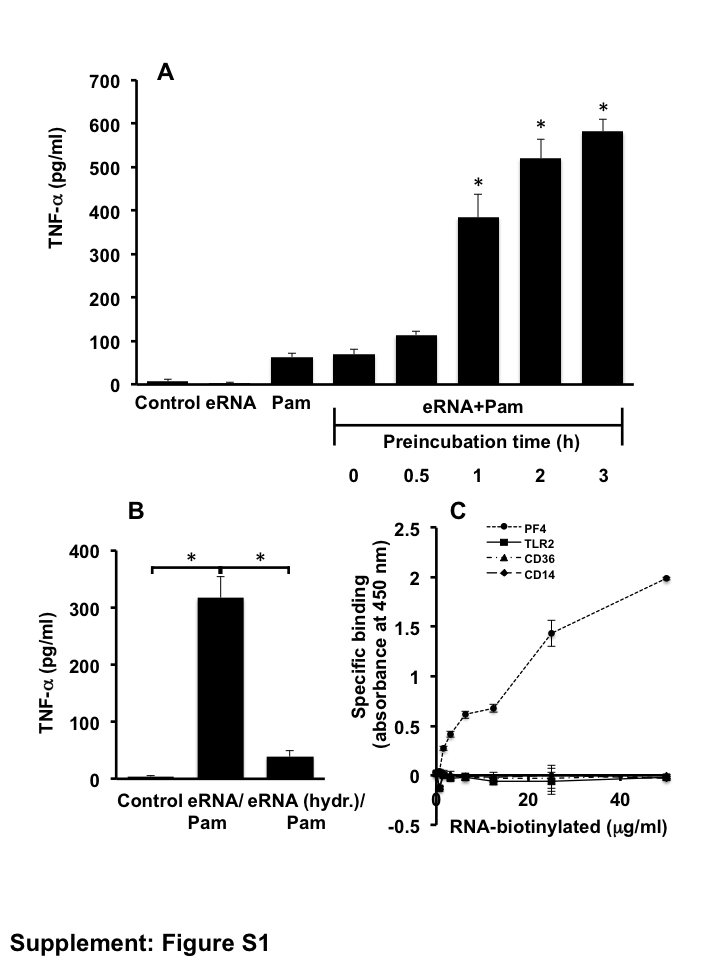

Supplement: S1 Fig — (A) Macrophages were treated with buffer (Control), Pam2CSK4 (Pam, 0.1 ng/ml), eRNA (10 μg/ml), or eRNA/Pam preincubated for different time intervalls prior to the treatment of cells for 2h. (B) Macrophages were treated with buffer (Control), 1h preincubated eRNA/Pam2CSK4 (eRNA/Pam), or eRNA predigested for 1h with RNase1 followed by 1h preincubation with Pam2CSK4 (eRNA(hydr.)/Pam). Supernatants of cells were analyzed for the release of TNF-α. Values are expressed as mean ± SEM. N = 3; *P < 0.05 between groups. (C) Binding of biotinylated RNA to immobilized PF4, TLR2, CD36, and CD14 was performed in a solid binding assay and data are corrected for unspecific binding to BSA. Data represent the mean ± SEM; N = 3. (TIFF) [file pone.0190002.s001.tiff]

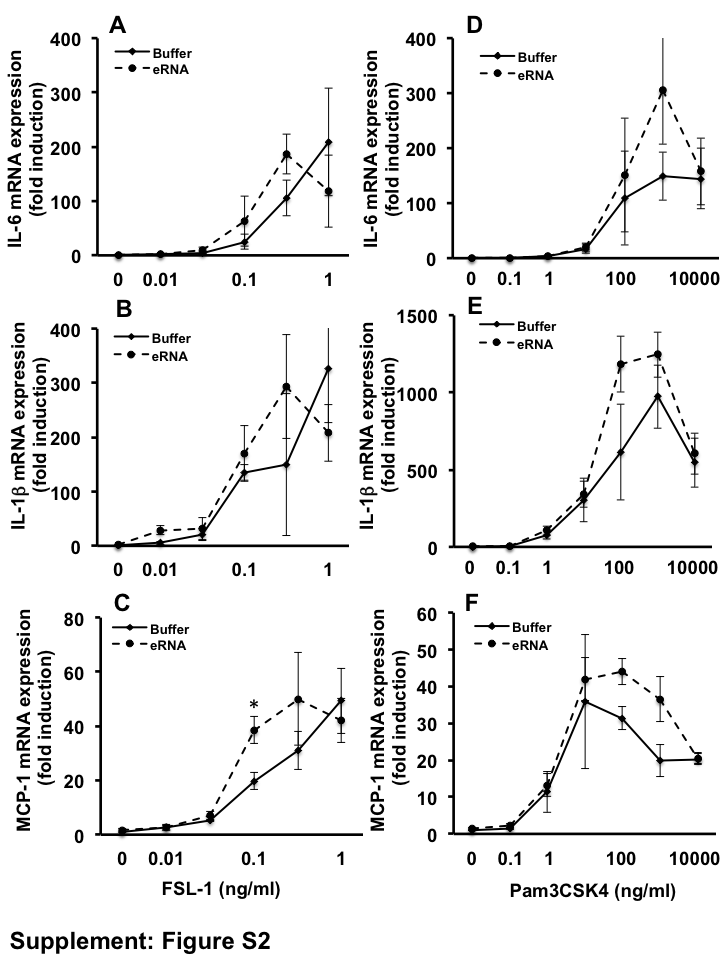

Supplement: S2 Fig — Macrophages were treated for 2 h with different concentrations of FSL-1 (A-C) or Pam3CSK4 (D-F) in the presence of eRNA (10 μg/ml) or buffer. Prior to cell stimulation, eRNA and FSL-1 or Pam2CSK4 were preincubated for 1 h at 37°C. mRNA expression of IL-6, IL-1β, and MCP1 was assessed from cell lysates by qRT-PCR. Values are expressed as mean ± SEM; N = 3–8; *P < 0.05 between groups. (TIFF) [file pone.0190002.s002.tiff]

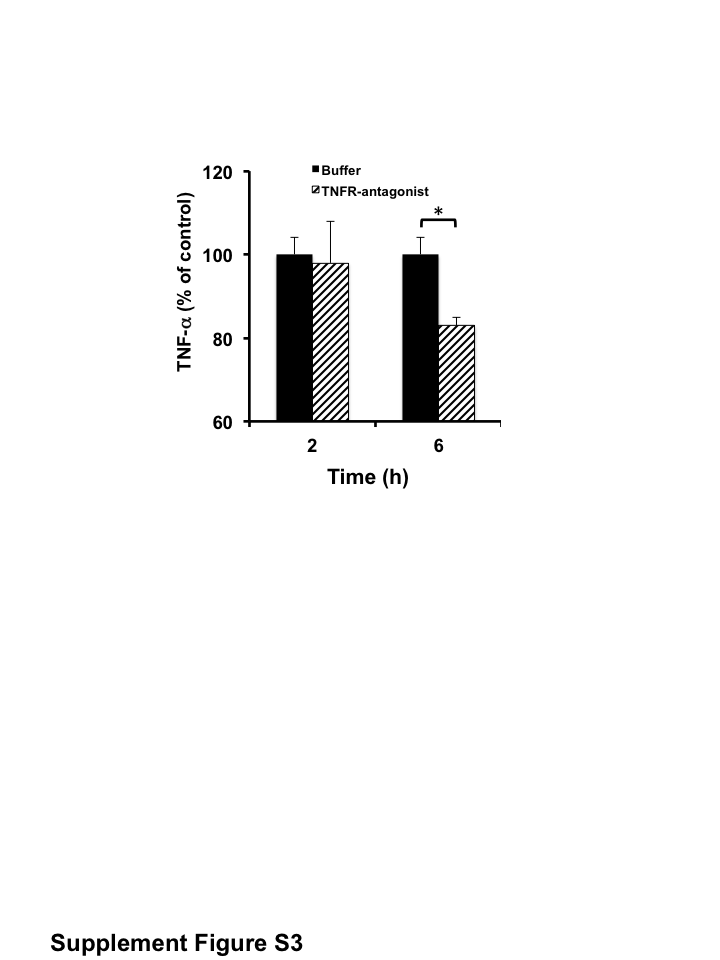

Supplement: S3 Fig — Macrophages were treated with eRNA (10 μg/ml)/Pam2CSK4 (0.1 ng/ml) (preincubated for 1 h at 37°C) for 2 h and 6 h in the presence of TNF-α receptor antagonist or buffer. Supernatants were analyzed for the release of TNF-α. Values determined in the absence of TNF-α receptor antagonist were set to 100%. Values are expressed as mean ± SEM; N = 3; *P < 0.05 between groups. (TIFF) [file pone.0190002.s003.tiff]

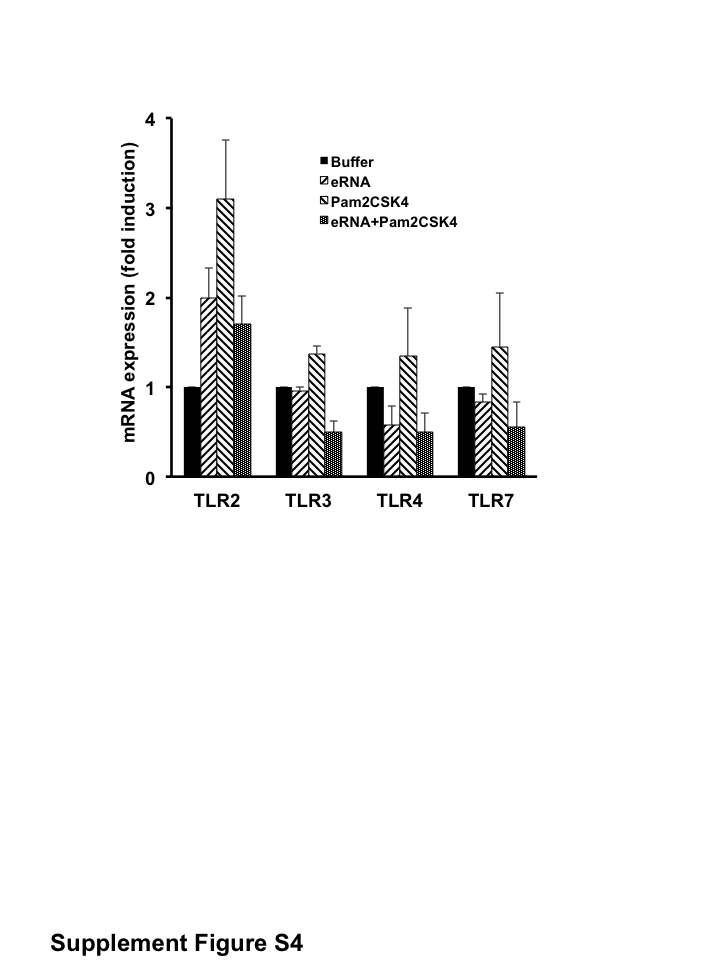

Supplement: S4 Fig — Macrophages were treated with eRNA (10 μg/ml), Pam2CSK4 (0.1 ng/ml), buffer, or the preincubated mixture for 2 h. mRNA expression of TLR2, TLR3, TLR4 and TLR7 was assessed from cell lysates by qRT-PCR. The expression of buffer-treated cells was set to one, and the data represent fold induction ± SEM; N = 3. (TIFF) [file pone.0190002.s004.tiff]
