# Supplementary material for: Self-extracellular RNA acts in synergy with exogenous danger signals to promote inflammation
Source: PLoS One. 2017 Dec 20;12(12):e0190002. doi: 10.1371/journal.pone.0190002 (PMC5738100; doi:10.1371/journal.pone.0190002)
Supplement: S1 Supplement — (DOCX) [file pone.0190002.s005.docx]

**Supporting information**

**Legends to Supplementary Figures**

**S1 Fig.:** **Synergistic activities of eRNA/Pam2CSK4 on TLR2 activation: Time-dependent preincubation.**

(A) Macrophages were treated with buffer (Control), Pam2CSK4 (Pam, 0.1 ng/ml), eRNA (10 μg/ml), or eRNA/Pam preincubated for different time intervalls prior to the treatment of cells for 2h. (B) Macrophages were treated with buffer (Control), 1h preincubated eRNA/Pam2CSK4 (eRNA/Pam), or eRNA predigested for 1h with RNase1 followed by 1h preincubation with Pam2CSK4 (eRNA(hydr.)/Pam). Supernatants of cells were analyzed for the release of TNF-α. Values are expressed as mean ± SEM. N = 3; *P < 0.05 between groups. (C) Binding of biotinylated RNA to immobilized PF4, TLR2, CD36, and CD14 was performed in a solid binding assay and data are corrected for unspecific binding to BSA. Data represent the mean ± SEM; N = 3.

**S2 Fig.: Sensitizing function of eRNA on TLR2 activation by TLR2-agonists FSL-1 and Pam3CSK4.**

Macrophages were treated for 2 h with different concentrations of FSL-1 (A-C) or Pam3CSK4 (D-F) in the presence of eRNA (10 μg/ml) or buffer. Prior to cell stimulation, eRNA and FSL-1 or Pam2CSK4 were preincubated for 1 h at 37°C. mRNA expression of IL-6, IL-1β, and MCP1 was assessed from cell lysates by qRT-PCR. Values are expressed as mean ± SEM; N = 3-8; *P < 0.05 between groups.

**S3 Fig.:** **Time dependent TNF-α release from macrophages treated with eRNA/Pam2CSK4.**

Macrophages were treated with eRNA (10 μg/ml)/Pam2CSK4 (0.1 ng/ml) (preincubated for 1 h at 37°C) for 2 h and 6 h in the presence of TNF-α receptor antagonist or buffer. Supernatants were analyzed for the release of TNF-α. Values determined in the absence of TNF-α-receptor antagonist were set to 100 %. Values are expressed as mean ± SEM; N = 3; *P < 0.05 between groups.

**S4 Fig.:** **Influence of eRNA and Pam2CSK4 on the expression of Toll-like receptors.**

Macrophages were treated with eRNA (10 μg/ml), Pam2CSK4 (0.1 ng/ml), buffer, or the preincubated mixture for 2 h. mRNA expression of TLR2, TLR3, TLR4 and TLR7 was assessed from cell lysates by qRT-PCR. The expression of buffer-treated cells was set to one, and the data represent fold induction ± SEM; N = 3.

**Figures and Data**

Fig. 1A

|  | Buffer | | | eRNA | | |
| --- | --- | --- | --- | --- | --- | --- |
| Pam2CSK4 | TNF-α |  |  | TNF-α |  |  |
| (ng/ml) | (pg/ml) | SEM | n | (pg/ml) | SEM | n |
| 0 | 0.0 | 0.0 | 9 | 0.4 | 1.2 | 9 |
| 0.01 | 0.0 | 0.0 | 6 | 16.5 | 7.4 | 6 |
| 0.03 | 0.4 | 0.3 | 3 | 33.9 | 5.9 | 3 |
| 0.10 | 10.0 | 4.0 | 5 | 349.4 | 11.4 | 4 |
| 0.30 | 81.5 | 13.2 | 3 | 347.1 | 57.1 | 3 |
| 1.00 | 333.4 | 34.8 | 12 | 392.8 | 39.9 | 12 |
| 10.00 | 181.2 | 21.7 | 17 | 256.7 | 29.2 | 17 |

Fig. 1B

|  | Buffer | | | Pam | | |
| --- | --- | --- | --- | --- | --- | --- |
| Treatments | TNF-α |  |  | TNF-α |  |  |
|  | (pg/ml) | SEM | n | (pg/ml) | SEM | n |
| eRNA | 0.2 | 0.2 | 3 | 343.0 | 13.0 | 3 |
| tRNA | 3.9 | 2.5 | 3 | 205.0 | 11.0 | 3 |
| eDNA | 6.2 | 5.0 | 3 | 92.1 | 10.8 | 3 |

Fig. 1C

|  | Buffer | | | eRNA | | |
| --- | --- | --- | --- | --- | --- | --- |
| Pam2CSK4 | TNF-α mRNA |  |  | TNF-α mRNA |  |  |
| (ng/ml) | fold induction | SEM | n | fold induction | SEM | n |
| 0.00 | 1.0 | 0.0 | 8 | 3.8 | 1.0 | 8 |
| 0.01 | 2.9 | 0.2 | 3 | 8.0 | 0.9 | 3 |
| 0.03 | 8.2 | 3.4 | 3 | 13.4 | 0.6 | 3 |
| 0.10 | 8.1 | 1.7 | 8 | 26.8 | 3.5 | 7 |
| 0.30 | 16.8 | 2.0 | 3 | 28.3 | 4.8 | 3 |
| 1.00 | 17.9 | 3.1 | 8 | 24.0 | 2.6 | 8 |
| 10.00 | 18.9 | 3.2 | 4 | 21.9 | 2.6 | 5 |

Fig. 1D

|  | Buffer | | | eRNA | | |
| --- | --- | --- | --- | --- | --- | --- |
| Pam2CSK4 | IL-6 mRNA |  |  | IL-6 mRNA |  |  |
| (ng/ml) | fold induction | SEM | n | fold induction | SEM | n |
| 0.00 | 1.0 | 0.0 | 8 | 1.2 | 0.2 | 8 |
| 0.01 | 1.2 | 0.1 | 3 | 3.8 | 1.4 | 3 |
| 0.03 | 7.4 | 5.6 | 3 | 11.3 | 2.3 | 3 |
| 0.10 | 8.3 | 3.4 | 8 | 112.2 | 29.6 | 6 |
| 0.30 | 33.8 | 10.0 | 3 | 195.1 | 69.9 | 3 |
| 1.00 | 95.0 | 29.3 | 8 | 144.6 | 32.7 | 8 |
| 10.00 | 96.9 | 20.3 | 4 | 125.6 | 18.8 | 5 |

Fig. 1E

|  | Buffer | | | eRNA | | |
| --- | --- | --- | --- | --- | --- | --- |
| Pam2CSK4 | IL-1β mRNA |  |  | IL-1β mRNA |  |  |
| (ng/ml) | fold induction | SEM | n | fold induction | SEM | n |
| 0.00 | 1.0 | 0.0 | 8 | 5.5 | 1.7 | 8 |
| 0.01 | 4.9 | 1.7 | 3 | 24.9 | 9.6 | 3 |
| 0.03 | 21.2 | 8.2 | 3 | 66.9 | 26.6 | 3 |
| 0.10 | 30.5 | 11.6 | 8 | 176.6 | 54.6 | 8 |
| 0.30 | 104.7 | 41.8 | 3 | 417.6 | 177.7 | 3 |
| 1.00 | 174.6 | 56.3 | 8 | 325.3 | 67.7 | 8 |
| 10.00 | 204.5 | 68.9 | 4 | 380.4 | 132.4 | 5 |

Fig. 1F

|  | Buffer | | | eRNA | | |
| --- | --- | --- | --- | --- | --- | --- |
| Pam2CSK4 | MCP-1 mRNA |  |  | MCP-1 mRNA |  |  |
| (ng/ml) | fold induction | SEM | n | fold induction | SEM | n |
| 0.00 | 1.0 | 0.0 | 8 | 1.2 | 0.1 | 8 |
| 0.01 | 1.3 | 0.0 | 3 | 2.4 | 0.5 | 3 |
| 0.03 | 2.9 | 1.3 | 3 | 6.6 | 0.6 | 3 |
| 0.10 | 3.3 | 0.9 | 9 | 26.8 | 3.7 | 6 |
| 0.30 | 14.1 | 3.0 | 3 | 49.1 | 7.9 | 3 |
| 1.00 | 40.1 | 6.3 | 7 | 44.8 | 7.7 | 8 |
| 10.00 | 26.4 | 4.9 | 5 | 28.5 | 3.2 | 6 |

Fig. 2A

|  | Buffer | | | Pam | | |
| --- | --- | --- | --- | --- | --- | --- |
| eRNA | TNF-α |  |  | TNF-α |  |  |
| (ng/ml) | (pg/ml) | SEM | n | (pg/ml) | SEM | n |
| 0 | 11.7 | 1.7 | 3 | 25.7 | 7.0 | 3 |
| 0.1 | 3.7 | 1.2 | 3 | 134.3 | 21.0 | 3 |
| 10 | 12.7 | 4.7 | 3 | 257.3 | 22.0 | 3 |
| 1000 | 1.7 | 1.7 | 3 | 295.0 | 38.0 | 3 |
| 10000 | 10 | 10 | 3 | 343.0 | 13.2 | 3 |

Fig. 2B

|  | Buffer | | | Pam | | |
| --- | --- | --- | --- | --- | --- | --- |
| eRNA | TNF-α mRNA |  |  | TNF-α mRNA |  |  |
| (ng/ml) | fold induction | SEM | n | fold induction | SEM | n |
| 0 | 1.0 | 0.0 | 3 | 3.4 | 0.5 | 3 |
| 0.1 | 1.1 | 0.1 | 3 | 11.4 | 1.4 | 3 |
| 10 | 0.9 | 0.1 | 3 | 17.1 | 1.4 | 3 |
| 1000 | 1.1 | 0.1 | 3 | 19.4 | 2.2 | 3 |
| 10000 | 2.1 | 0.6 | 3 | 30.7 | 8.2 | 3 |

Fig. 2C

|  | Buffer | | | Pam | | |
| --- | --- | --- | --- | --- | --- | --- |
| eRNA | IL-6 mRNA |  |  | IL-6 mRNA |  |  |
| (ng/ml) | fold induction | SEM | n | fold induction | SEM | n |
| 0 | 1.0 | 0.0 | 3 | 2.0 | 0.9 | 3 |
| 0.1 | 0.8 | 0.3 | 3 | 21.0 | 14.7 | 3 |
| 10 | 0.5 | 0.2 | 3 | 29.4 | 10.4 | 3 |
| 1000 | 0.6 | 0.3 | 3 | 25.4 | 10.1 | 3 |
| 10000 | 1.2 | 0.7 | 3 | 40.3 | 12.2 | 3 |

Fig. 2D

|  | Buffer | | | Pam | | |
| --- | --- | --- | --- | --- | --- | --- |
| eRNA | IL-1β mRNA |  |  | IL-1β mRNA |  |  |
| (ng/ml) | fold induction | SEM | n | fold induction | SEM | n |
| 0 | 1.0 | 0.0 | 3 | 6.6 | 0.8 | 3 |
| 0.1 | 0.8 | 0.2 | 3 | 34.0 | 6.9 | 3 |
| 10 | 0.9 | 0.2 | 3 | 96.4 | 22.4 | 3 |
| 1000 | 0.8 | 0.1 | 3 | 97.2 | 12.5 | 3 |
| 10000 | 1.5 | 0.0 | 3 | 201.5 | 70.3 | 3 |

Fig. 2E

|  | Buffer | | | Pam | | |
| --- | --- | --- | --- | --- | --- | --- |
| eRNA | MCP-1 mRNA |  |  | MCP-1 mRNA |  |  |
| (ng/ml) | fold induction | SEM | n | fold induction | SEM | n |
| 0 | 1.0 | 0.0 | 3 | 1.3 | 0.3 | 3 |
| 0.1 | 1.2 | 0.3 | 3 | 6.8 | 1.5 | 3 |
| 10 | 0.9 | 0.2 | 3 | 18.7 | 3.9 | 3 |
| 1000 | 1.1 | 0.3 | 3 | 26.1 | 7.1 | 3 |
| 10000 | 1.7 | 0.4 | 3 | 55.1 | 23.6 | 3 |

Fig. 3A

|  | Buffer | | | eRNA | | |
| --- | --- | --- | --- | --- | --- | --- |
| FSL | TNF-α |  |  | TNF-α |  |  |
| (ng/ml) | (pg/ml) | SEM | n | (pg/ml) | SEM | n |
| 0.00 | 0.0 | 0.0 | 8 | 0.0 | 0.0 | 8 |
| 0.01 | 8.6 | 2.5 | 5 | 31.2 | 8.8 | 5 |
| 0.03 | 52.1 | 18.3 | 5 | 127.5 | 40.2 | 5 |
| 0.10 | 169.5 | 17.3 | 8 | 332.9 | 33.9 | 8 |
| 0.30 | 289.0 | 24.1 | 5 | 382.9 | 14.3 | 5 |
| 1.00 | 322.4 | 16.6 | 8 | 289.4 | 17.7 | 8 |

Fig. 3B

|  | Buffer | | | eRNA | | |
| --- | --- | --- | --- | --- | --- | --- |
| Pam3CSK4 | TNF-α |  |  | TNF-α |  |  |
| (ng/ml) | (pg/ml) | SEM | n | (pg/ml) | SEM | n |
| 0 | 0.0 | 10.0 | 3 | 0.0 | 0.0 | 3 |
| 0.1 | 0.0 | 9.6 | 3 | 0.0 | 0.0 | 3 |
| 1 | 80.1 | 7.0 | 3 | 103.8 | 10.6 | 3 |
| 10 | 260.4 | 0.0 | 3 | 420.2 | 26.6 | 3 |
| 100 | 373.5 | 0.0 | 5 | 504.6 | 23.7 | 5 |
| 1000 | 245.9 | 35.4 | 3 | 419.0 | 49.0 | 3 |
| 10000 | 169.2 | 25.7 | 3 | 234.0 | 42.0 | 3 |

Fig. 3C

|  | Buffer | | | eRNA | | |
| --- | --- | --- | --- | --- | --- | --- |
| FSL | TNF-α mRNA |  |  | TNF-α mRNA |  |  |
| (ng/ml) | fold induction | SEM | n | fold induction | SEM | n |
| 0.00 | 1.0 | 0.0 | 5 | 1.9 | 0.3 | 8 |
| 0.01 | 3.6 | 1.6 | 4 | 6.3 | 2.7 | 4 |
| 0.03 | 7.5 | 3.4 | 3 | 8.7 | 4.0 | 3 |
| 0.10 | 18.9 | 4.1 | 7 | 33.4 | 0.9 | 4 |
| 0.30 | 17.9 | 9.6 | 3 | 28.8 | 2.5 | 3 |
| 1.00 | 30.2 | 4.1 | 7 | 24.6 | 2.5 | 7 |

Fig. 3D

|  | Buffer | | | eRNA | | |
| --- | --- | --- | --- | --- | --- | --- |
| Pam3CSK4 | TNF-α mRNA |  |  | TNF-α mRNA |  |  |
| (ng/ml) | fold induction | SEM | n | fold induction | SEM | n |
| 0 | 1.0 | 0.0 | 3 | 2.1 | 0.1 | 9 |
| 0.1 | 4.2 | 0.8 | 3 | 9.3 | 1.0 | 3 |
| 1 | 19.7 | 2.2 | 3 | 23.4 | 1.8 | 3 |
| 10 | 31.8 | 5.8 | 3 | 40.0 | 0.7 | 3 |
| 100 | 32.7 | 4.2 | 5 | 40.0 | 6.8 | 5 |
| 1000 | 38.2 | 5.4 | 3 | 45.9 | 7.9 | 3 |
| 10000 | 48.2 | 8.8 | 3 | 37.4 | 5.7 | 3 |

Fig. 4A

|  | Buffer | | | eRNA | | | Pam | | | eRNA+Pam | | |
| --- | --- | --- | --- | --- | --- | --- | --- | --- | --- | --- | --- | --- |
| time | TNF-α |  |  | TNF-α |  |  | TNF-α |  |  | TNF-α |  |  |
| (h) | (pg/m) | SEM | n | (pg/ml) | SEM | n | (pg/ml) | SEM | n | (pg/ml) | SEM | n |
| 1 | 0.0 | 0.0 | 3 | 0.0 | 0.0 | 3 | 39.7 | 20.9 | 3 | 275.0 | 25.1 | 3 |
| 2 | 0.2 | 0.2 | 3 | 37.4 | 22.4 | 3 | 54.3 | 9.3 | 3 | 457.7 | 29.2 | 3 |
| 6 | 0.0 | 0.0 | 3 | 34.5 | 17.8 | 3 | 79.7 | 3.6 | 3 | 1054.0 | 162.7 | 3 |
| 12 | 5.4 | 2.9 | 3 | 31.4 | 17.8 | 3 | 83.6 | 0.9 | 3 | 867.9 | 30.7 | 3 |
| 24 | 1.7 | 1.0 | 3 | 39.3 | 14.8 | 3 | 60.7 | 9.6 | 3 | 833.1 | 177.9 | 3 |

Fig. 4B

|  | Buffer | | | eRNA | | | Pam | | | eRNA+Pam | | |
| --- | --- | --- | --- | --- | --- | --- | --- | --- | --- | --- | --- | --- |
| time | IL-6 |  |  | IL-6 |  |  | IL-6 |  |  | IL-6 |  |  |
| (h) | (pg/ml) | SEM | n | (pg/ml) | SEM | n | (pg/ml) | SEM | n | (pg/ml) | SEM | n |
| 1 | 0.0 | 0.0 | 3 | 0.0 | 0.0 | 3 | 0.0 | 0.0 | 3 | 0.0 | 0.0 | 3 |
| 2 | 0.0 | 0.0 | 3 | 0.0 | 0.0 | 3 | 0.0 | 0.0 | 3 | 0.0 | 0.0 | 3 |
| 6 | 13.3 | 13.3 | 3 | 0.0 | 0.0 | 3 | 16.3 | 11.3 | 3 | 831.3 | 99.8 | 3 |
| 12 | 8.0 | 4.9 | 3 | 6.0 | 6.0 | 3 | 31.0 | 30.5 | 3 | 1655.0 | 156.9 | 3 |
| 24 | 19.3 | 19.3 | 3 | 4.3 | 4.3 | 3 | 4.0 | 4.0 | 3 | 1693.0 | 201.9 | 3 |

Fig. 4C

|  | Buffer | | | eRNA | | | Pam | | | eRNA+Pam | | |
| --- | --- | --- | --- | --- | --- | --- | --- | --- | --- | --- | --- | --- |
| time | TNF-α mRNA |  |  | TNF-α mRNA |  |  | TNF-α mRNA |  |  | TNF-α  mRNA |  |  |
| (h) | fold induction | SEM | n | fold induction | SEM | n | fold induction | SEM | n | fold  induction | SEM | n |
| 1 | 1.0 | 0.0 | 3 | 2.0 | 0.2 | 3 | 17.0 | 6.1 | 3 | 27.0 | 11.4 | 3 |
| 2 | 1.0 | 0.0 | 3 | 6.7 | 3.6 | 3 | 7.3 | 1.1 | 3 | 24.0 | 0.1 | 3 |
| 6 | 1.0 | 0.0 | 3 | 1.6 | 0.2 | 3 | 5.3 | 0.5 | 3 | 38.2 | 0.7 | 3 |
| 12 | 1.0 | 0.0 | 3 | 1.0 | 0.2 | 3 | 1.6 | 0.2 | 3 | 8.1 | 2.5 | 3 |
| 24 | 1.0 | 0.0 | 3 | 0.7 | 0.1 | 3 | 0.6 | 0.0 | 3 | 2.0 | 0.4 | 3 |

Fig. 4D

|  | Buffer | | | eRNA | | | Pam | | | eRNA+Pam | | |
| --- | --- | --- | --- | --- | --- | --- | --- | --- | --- | --- | --- | --- |
| time | IL-6 mRNA |  |  | IL-6 mRNA |  |  | IL-6 mRNA |  |  | IL-6 mRNA |  |  |
| (h) | fold induction | SEM | n | fold induction | SEM | n | fold induction | SEM | n | fold induction | SEM | n |
| 1 | 1.0 | 0.0 | 3 | 1.2 | 0.3 | 3 | 13.1 | 7.6 | 3 | 63.6 | 12.5 | 3 |
| 2 | 1.0 | 0.0 | 3 | 5.5 | 2.7 | 3 | 20.3 | 6.1 | 3 | 547.0 | 169.2 | 3 |
| 6 | 1.0 | 0.0 | 3 | 1.2 | 0.3 | 3 | 8.1 | 0.5 | 3 | 2497.8 | 1030.6 | 3 |
| 12 | 1.0 | 0.0 | 3 | 0.7 | 0.1 | 3 | 2.2 | 1.0 | 3 | 709.1 | 343.0 | 3 |
| 24 | 1.0 | 0.0 | 3 | 1.6 | 1.1 | 3 | 0.6 | 0.3 | 3 | 196.0 | 31.0 | 3 |

Fig. 4E

|  | Buffer | | | eRNA | | | Pam | | | eRNA+Pam | | |
| --- | --- | --- | --- | --- | --- | --- | --- | --- | --- | --- | --- | --- |
| time | IL-1β mRNA |  |  | IL-1β mRNA |  |  | IL-1β mRNA |  |  | IL-1β mRNA |  |  |
| (h) | fold induction | SEM | n | fold induction | SEM | n | fold induction | SEM | n | fold induction | SEM | n |
| 1 | 1.0 | 0.0 | 3 | 1.4 | 0.0 | 3 | 72.9 | 4.2 | 3 | 509.4 | 152.4 | 3 |
| 2 | 1.0 | 0.0 | 3 | 22.5 | 11.8 | 3 | 30.5 | 2.9 | 3 | 362.7 | 79.1 | 3 |
| 6 | 1.0 | 0.0 | 3 | 0.9 | 0.2 | 3 | 8.7 | 3.0 | 3 | 557.6 | 114.9 | 3 |
| 12 | 1.0 | 0.0 | 3 | 0.5 | 0.1 | 3 | 0.5 | 0.1 | 3 | 18.3 | 9.2 | 3 |
| 24 | 1.0 | 0.0 | 3 | 1.5 | 0.5 | 3 | 1.9 | 0.3 | 3 | 1.2 | 0.3 | 3 |

Fig. 4F

|  | Buffer | | | eRNA | | | Pam | | | eRNA+Pam | | |
| --- | --- | --- | --- | --- | --- | --- | --- | --- | --- | --- | --- | --- |
| time | MCP-1 mRNA |  |  | MCP-1 mRNA |  |  | MCP-1 mRNA |  |  | MCP-1 mRNA |  |  |
| (h) | fold induction | SEM | n | fold induction | SEM | n | fold induction | SEM | n | fold induction | SEM | n |
| 1 | 1.0 | 0.0 | 3 | 1.2 | 0.3 | 3 | 4.7 | 1.6 | 3 | 16.4 | 5.4 | 3 |
| 2 | 1.0 | 0.0 | 3 | 6.2 | 2.9 | 3 | 5.1 | 2.0 | 3 | 58.8 | 21.8 | 3 |
| 6 | 1.0 | 0.0 | 3 | 2.8 | 0.9 | 3 | 6.2 | 1.2 | 3 | 169.0 | 66.3 | 3 |
| 12 | 1.0 | 0.0 | 3 | 1.1 | 0.4 | 3 | 2.5 | 1.2 | 3 | 86.7 | 44.4 | 3 |
| 24 | 1.0 | 0.0 | 3 | 0.7 | 0.2 | 3 | 0.4 | 0.1 | 3 | 9.0 | 3.9 | 3 |

Fig. 5A

| Treatments | TNF-α protein | | |
| --- | --- | --- | --- |
|  | (%) | SEM | n |
| C | 100.0 | 6.4 | 3 |
| anti-TLR2 | 3.8 | 2.3 | 3 |
| TAPI | 21.1 | 1.2 | 3 |
| Bay | 0.3 | 0.3 | 3 |
| PD98059 | 51.1 | 2.3 | 3 |
| SB203580 | 29.1 | 0.9 | 3 |

Fig. 5B

| Treatments | TNF-α mRNA | | |
| --- | --- | --- | --- |
|  | (%) | SEM | n |
| C | 100.0 | 3.8 | 3 |
| anti-TLR2 | 37.5 | 2.3 | 3 |
| TAPI | 113.9 | 1.2 | 3 |
| Bay | 4.2 | 0.3 | 3 |
| PD98059 | 80.8 | 2.3 | 3 |
| SB203580 | 105.0 | 0.9 | 3 |

Fig. 5C

| Treatments | IL-6 mRNA | | |
| --- | --- | --- | --- |
|  | (%) | SEM | n |
| C | 100.0 | 0.0 | 3 |
| anti-TLR2 | 6.6 | 0.9 | 3 |
| TAPI | 88.1 | 8.4 | 3 |
| Bay | 1.7 | 0.9 | 3 |
| PD98059 | 115.5 | 23.6 | 3 |
| SB203580 | 46.0 | 1.2 | 3 |

Fig. 5D

| Treatments | IL-1β mRNA | | |
| --- | --- | --- | --- |
|  | (%) | SEM | n |
| C | 100.0 | 0.0 | 3 |
| anti-TLR2 | 14.1 | 1.5 | 3 |
| TAPI | 164.3 | 42.1 | 3 |
| Bay | 0.8 | 0.4 | 3 |
| PD98059 | 57.9 | 7.6 | 3 |
| SB203580 | 27.8 | 4.7 | 3 |

Fig. 5E

| Treatments | MCP-1 mRNA | | |
| --- | --- | --- | --- |
|  | (%) | SEM | n |
| C | 100.0 | 0.0 | 3 |
| anti-TLR2 | 10.0 | 0.9 | 3 |
| TAPI | 267.7 | 108.1 | 3 |
| Bay | 11.2 | 5.2 | 3 |
| PD98059 | 62.1 | 1.6 | 3 |
| SB203580 | 140.5 | 9.2 | 3 |

Fig. 6A

|  | Buffer | | | eRNA | | |
| --- | --- | --- | --- | --- | --- | --- |
| LPS | TNF-α |  |  | TNF-α |  |  |
| (ng/ml) | (pg/ml) | SEM | n | (pg/ml) | SEM | n |
| 0 | 0.0 | 0.0 | 3 | 0.0 | 0.0 | 3 |
| 0.1 | 12.2 | 10.0 | 3 | 54.5 | 2.6 | 3 |
| 1 | 440.8 | 66.9 | 3 | 678.7 | 64.7 | 3 |
| 10 | 1092.7 | 23.7 | 3 | 1021.1 | 42.0 | 3 |

Fig. 6B

|  | Buffer | | | eRNA | | |
| --- | --- | --- | --- | --- | --- | --- |
| R848 | TNF-α |  |  | TNF-α |  |  |
| (ng/ml) | (pg/ml) | SEM | n | (pg/ml) | SEM | n |
| 0 | 0.0 | 0.0 | 3 | 0 | 0.0 | 3 |
| 0.1 | 0.0 | 0.0 | 3 | 0 | 0.0 | 3 |
| 1 | 0.0 | 0.0 | 3 | 0.4 | 0.3 | 3 |
| 10 | 83.3 | 21.8 | 3 | 89.2 | 27.9 | 3 |
| 100 | 530.8 | 29.0 | 3 | 557.0 | 15.3 | 3 |

Fig. 6C

|  | Buffer | | | eRNA | | |
| --- | --- | --- | --- | --- | --- | --- |
| poly IC | TNF-α |  |  | TNF-α |  |  |
| (ng/ml) | (pg/ml) | SEM | n | (pg/ml) | SEM | n |
| 0 | 0 | 0.0 | 3 | 0 | 0.0 | 3 |
| 1 | 0 | 0.0 | 3 | 0 | 0.0 | 3 |
| 10 | 0 | 0.0 | 3 | 0 | 0.0 | 3 |
| 100 | 0 | 0.0 | 3 | 0 | 0.0 | 3 |
| 1000 | 38 | 16.2 | 3 | 15 | 12.1 | 3 |

Fig. 6D

|  | Buffer | | | eRNA | | |
| --- | --- | --- | --- | --- | --- | --- |
| LPS | TNF-α mRNA |  |  | TNF-α mRNA |  |  |
| (ng/ml) | fold induction | SEM | n | fold induction | SEM | n |
| 0 | 1.0 | 0.0 | 3 | 6.3 | 0.7 | 3 |
| 0.1 | 10.2 | 0.9 | 3 | 13.6 | 1.4 | 3 |
| 1 | 29.1 | 6.0 | 3 | 45.0 | 9.3 | 3 |
| 10 | 65.2 | 10.3 | 3 | 63.5 | 23.0 | 3 |

Fig. 6E

|  | Buffer | | | eRNA | | |
| --- | --- | --- | --- | --- | --- | --- |
| R848 | TNF-α mRNA |  |  | TNF-α mRNA |  |  |
| (ng/ml) | fold induction | SEM | n | fold induction | SEM | n |
| 0 | 1.0 |  | 3 | 2.2 | 0.3 | 3 |
| 0.1 | 0.9 | 0.1 | 3 | 1.6 | 0.4 | 3 |
| 1 | 2.5 | 0.2 | 3 | 3.2 | 0.1 | 3 |
| 10 | 41.5 | 8.3 | 3 | 51.8 | 6.0 | 3 |
| 100 | 67.2 | 6.8 | 3 | 84.0 | 8.0 | 3 |

Fig. 6F

|  | Buffer | | | eRNA | | |
| --- | --- | --- | --- | --- | --- | --- |
| poly IC | TNF-α mRNA |  |  | TNF-α mRNA |  |  |
| (ng/ml) | fold induction | SEM | n | fold induction | SEM | n |
| 0 | 1 |  | 3 | 3.7 | 1.1 | 3 |
| 1 | 1.1 | 0.0 | 3 | 3 | 0.8 | 3 |
| 10 | 1.5 | 0.5 | 3 | 4.2 | 3.0 | 3 |
| 100 | 10.7 | 3.4 | 3 | 4.4 | 2.0 | 3 |
| 1000 | 13.6 | 2.1 | 3 | 6.2 | 2.2 | 3 |

Supplement

Fig. S1 A

| Treatments | Incubation- | TNF-α | | |
| --- | --- | --- | --- | --- |
|  | time |  |  |  |
|  | (h) | (pg/ml) | SEM | n |
| Control |  | 6.7 | 4.0 | 3 |
| eRNA |  | 3.1 | 1.7 | 3 |
| Pam |  | 63.5 | 6.4 | 3 |
| eRNA+Pam | 0 | 68.7 | 9.2 | 3 |
|  | 0.5 | 114.1 | 6.9 | 3 |
|  | 1 | 384.6 | 43.9 | 3 |
|  | 2 | 520.4 | 35.2 | 3 |
|  | 3 | 582.0 | 23.7 | 3 |

Fig. S1 B

|  | TNF-α |  |  |
| --- | --- | --- | --- |
|  | (pg/ml) | SEM | n |
| Control | 4.3 | 0.3 | 3 |
| eRNA/Pam | 317.0 | 26.0 | 3 |
| eRNA (hydr.)/ Pam | 38.7 | 7.3 | 3 |

Fig. S1 C

| biot. RNA | absorbance (450 nm) | | absorbance (450 nm) | | absorbance (450 nm) | | absorbance (450 nm) | |
| --- | --- | --- | --- | --- | --- | --- | --- | --- |
| (μg/ml) | PF4 | SEM | TLR2 | SEM | CD36 | SEM | CD14 | SEM |
| 50 | 1.99 | 0.01 | -0.02 | 0.01 | 0.00 | 0.00 | -0.03 | 0.00 |
| 25 | 1.43 | 0.13 | -0.06 | 0.00 | -0.03 | 0.01 | 0.00 | 0.03 |
| 12.5 | 0.68 | 0.04 | -0.06 | 0.01 | -0.03 | 0.01 | -0.02 | 0.05 |
| 6.25 | 0.61 | 0.04 | -0.02 | 0.01 | -0.02 | 0.01 | -0.01 | 0.01 |
| 3.125 | 0.41 | 0.03 | -0.03 | 0.00 | 0.01 | 0.02 | -0.01 | 0.02 |
| 1.563 | 0.27 | 0.02 | 0.00 | 0.00 | 0.01 | 0.01 | 0.02 | 0.03 |
| 0.781 | 0.05 | 0.02 | -0.13 | 0.00 | -0.12 | 0.02 | -0.12 | 0.02 |
| 0 | 0.02 | 0.01 | 0.02 | 0.04 | 0.04 | 0.01 | 0.00 | 0.00 |

n = 3 each

Fig. S2 A

|  | Buffer | | | eRNA | | |
| --- | --- | --- | --- | --- | --- | --- |
| FSL | IL-6 mRNA |  |  | IL-6 mRNA |  |  |
| (ng/ml) | fold induction | SEM | n | fold induction | SEM | n |
| 0.00 | 1.0 | 0.0 | 5 | 1.0 | 0.3 | 8 |
| 0.01 | 2.4 | 0.8 | 7 | 2.4 | 0.8 | 7 |
| 0.03 | 4.9 | 2.3 | 3 | 9.0 | 6.4 | 3 |
| 0.10 | 25.2 | 14.0 | 7 | 62.9 | 45.5 | 7 |
| 0.30 | 106.3 | 33.0 | 3 | 187.2 | 37.0 | 3 |
| 1.00 | 209.3 | 98.7 | 6 | 118.0 | 65.7 | 7 |

Fig. S2 B

|  | Buffer | | | eRNA | | |
| --- | --- | --- | --- | --- | --- | --- |
| FSL | IL-1β mRNA |  |  | IL-1β mRNA |  |  |
| (ng/ml) | fold induction | SEM | n | fold induction | SEM | n |
| 0.00 | 1.0 |  | 5 | 1.7 | 0.7 | 8 |
| 0.01 | 5.1 | 3 | 4 | 28.5 | 8.3 | 7 |
| 0.03 | 20.8 | 11 | 3 | 31.7 | 21 | 3 |
| 0.10 | 135.0 | 14 | 4 | 170 | 51 | 4 |
| 0.30 | 150.0 | 131 | 4 | 294 | 96 | 4 |
| 1.00 | 327.0 | 100 | 7 | 208 | 52 | 7 |

Fig. S2 C

|  | Buffer | | | eRNA | | |
| --- | --- | --- | --- | --- | --- | --- |
| FSL | MCP-1 mRNA |  |  | MCP-1 mRNA |  |  |
| (ng/ml) | fold induction | SEM | n | fold induction | SEM | n |
| 0.00 | 1.0 |  | 5 | 1.7 | 0.7 | 8 |
| 0.01 | 2.5 | 0.8 | 4 | 2.8 | 1.1 | 7 |
| 0.03 | 5.1 | 0.4 | 3 | 7.0 | 1.7 | 3 |
| 0.10 | 19.8 | 3.4 | 4 | 38.6 | 5.1 | 4 |
| 0.30 | 31.0 | 6.6 | 4 | 50.0 | 17.0 | 4 |
| 1.00 | 49.3 | 12.5 | 7 | 42.1 | 8.1 | 7 |

Fig. S2 D

|  | Buffer | | | eRNA | | |
| --- | --- | --- | --- | --- | --- | --- |
| Pam3CSK4 | IL-6 mRNA |  |  | IL-6 mRNA |  |  |
| (ng/ml) | fold induction | SEM | n | fold induction | SEM | n |
| 0 | 1.0 | 0.0 | 3 | 0.8 | 0.2 | 9 |
| 0.1 | 0.6 | 0.1 | 3 | 0.7 | 0.3 | 3 |
| 1 | 3.2 | 1.6 | 3 | 3.3 | 1.3 | 3 |
| 10 | 17.3 | 9.0 | 3 | 20.0 | 7.9 | 3 |
| 100 | 109.3 | 85.2 | 5 | 151.2 | 103.3 | 5 |
| 1000 | 149.3 | 44.0 | 3 | 305.3 | 98.5 | 3 |
| 10000 | 144.7 | 54.7 | 3 | 157.7 | 60.5 | 3 |

Fig. S2 E

|  | Buffer | | | eRNA | | |
| --- | --- | --- | --- | --- | --- | --- |
| Pam3CSK4 | IL-1β mRNA |  |  | IL-1β mRNA |  |  |
| (ng/ml) | fold induction | SEM | n | fold induction | SEM | n |
| 0 | 1.0 | 0.0 | 3 | 1.3 | 0.1 | 9 |
| 0.1 | 3.6 | 1.8 | 3 | 7.3 | 1.4 | 3 |
| 1 | 78.4 | 27.1 | 3 | 105.8 | 28.6 | 3 |
| 10 | 304.7 | 141.6 | 3 | 340.1 | 84.3 | 3 |
| 100 | 614.8 | 310.4 | 5 | 1184.0 | 380.3 | 5 |
| 1000 | 973.3 | 405.7 | 3 | 1246.0 | 347.9 | 3 |
| 10000 | 545.7 | 158.5 | 3 | 605.0 | 133.6 | 3 |

Fig. S2 F

|  | Buffer | | | eRNA | | |
| --- | --- | --- | --- | --- | --- | --- |
| Pam3CSK4 | MCP-1 mRNA |  |  | MCP-1 mRNA |  |  |
| (ng/ml) | fold induction | SEM | n | fold induction | SEM | n |
| 0 | 1.0 | 0.0 | 3 | 1.1 | 0.2 | 9 |
| 0.1 | 1.5 | 0.2 | 3 | 2.3 | 0.6 | 3 |
| 1 | 11.4 | 5.5 | 3 | 13.2 | 3.0 | 3 |
| 10 | 35.9 | 18.3 | 3 | 41.9 | 5.8 | 3 |
| 100 | 31.4 | 3.2 | 5 | 44.0 | 3.5 | 4 |
| 1000 | 19.9 | 4.4 | 3 | 36.5 | 6.1 | 3 |
| 10000 | 20.27 | 1.4 | 3 | 20.5 | 1.5 | 3 |

Fig. S3

| time (h) | 2 | | | 6 | | |
| --- | --- | --- | --- | --- | --- | --- |
|  | TNF-α |  |  | TNF-α |  |  |
|  | (% ) | SEM | n | (% ) | SEM | n |
| Buffer | 100.0 | 4.1 | 3 | 100.0 | 4.0 | 3 |
| TNFR- antagonist | 98.2 | 10.2 | 3 | 83.1 | 2.0 | 3 |

Fig. S4

| TLR2 | | | TLR3 | | | TLR4 | | | TLR7 | | |
| --- | --- | --- | --- | --- | --- | --- | --- | --- | --- | --- | --- |
| mRNA |  |  | mRNA |  |  | mRNA |  |  | mRNA |  |  |
| fold induction | SEM | n | fold induction | SEM | n | fold induction | SEM | n | fold induction | SEM | n |
| 1.0 | 0.0 | 3 | 1.0 | 0.0 | 3 | 1.0 | 0.0 | 3 | 1.0 | 0.0 | 3 |
| 2.0 | 0.3 | 3 | 1.0 | 0.0 | 3 | 0.6 | 0.2 | 3 | 0.8 | 0.1 | 3 |
| 3.1 | 0.7 | 3 | 1.4 | 0.1 | 3 | 1.3 | 0.5 | 3 | 1.5 | 0.6 | 3 |
| 1.7 | 0.3 | 3 | 0.5 | 0.1 | 3 | 0.5 | 0.2 | 3 | 0.6 | 0.3 | 3 |
